# Supplementary material for: Azido-Ceramides, a Tool to Analyse SARS-CoV-2 Replication and Inhibition—SARS-CoV-2 Is Inhibited by Ceramides
Source: Int J Mol Sci. 2023 Apr 14;24(8):7281. doi: 10.3390/ijms24087281 (PMC10138768; doi:10.3390/ijms24087281)
Supplement: Supplementary file 1 [file ijms-24-07281-s001.zip › ijms-2313778-supplementary.pdf]

Supporting information

Azido-Ceramides, a Tool to Analyse SARS-CoV-2 Replication and Inhibition—SARS-CoV-2 Is Inhibited by Ceramides

Daniela Brenner [1,†], Nina Geiger [2,†], Jan Schlegel [3,‡], Viktoria Diesendorf [2], Louise Kersting [1], Julian Fink [1], Linda Stelz [3], Sibylle Schneider-Schaulies [2], Markus Sauer [3], Jochen Bodem [2,*] and Jürgen Seibel [1,*]

[1] Institute of Organic Chemistry, Julius-Maximilians-Universität Würzburg, 97074 Würzburg, Germany; daniela.brenner@uni-wuerzburg.de (D.B.); louise.kersting@uni-wuerzburg.de (L.K.)
[2] Institute of Virology and Immunobiology, Julius-Maximilians-Universität Würzburg, 97078 Würzburg, Germany; nina.geiger@stud-mail.uni-wuerzburg.de (N.G.); viktoria.diesendorf@stud-mail.uni-wuerzburg.de (V.D.)
[3] Department of Biotechnology and Biophysics, Julius-Maximilians-Universität Würzburg, 97074 Würzburg, Germany; jan.schlegel@scilifelab.se (J.S.); m.sauer@uni-wuerzburg.de (M.S.)
* Correspondence: jochen.bodem@uni-wuerzburg.de (J.B.); seibel@chemie.uni-wuerzburg.de (J.S.)
† These authors contributed equally to this work.
‡ Current Address: Department of Women's and Children's Health, Karolinska Institute, SciLifeLab, Tomtebodavägen 23B, Solna, 171 65 Stockholm, Sweden

1. Oligonucleotide sequences for FISH labelling

CGCCTAAGTCAAATGACTTTAGATCGGCGCTTACACTCGGACCTCGTCGACATGCATT
GGTCCACCAAACGTAATGCGGGGTGCATTACACTCGGACCTCGTCGACATGCATT
ATCTTGAAGTTTTCCAAGTGCACTTGCTGTGTTACACTCGGACCTCGTCGACATGCATT
TAACATGACCATGAGGTGCAGTTCGAGCATTTACACTCGGACCTCGTCGACATGCATT
TGTGATCAACCTATCAATTTGCACTTCAGCTTACACTCGGACCTCGTCGACATGCATT
GTTGTTTTGATCGCGCCCCACTGCGTTTACACTCGGACCTCGTCGACATGCATT
TTGAACTGTTGCGACTACGTGATGAGGAACGATTACACTCGGACCTCGTCGACATGCATT
AAGTGTCTCACCACTACGACCGTACTGAATTACACTCGGACCTCGTCGACATGCATT
CGCCTTGTCCTCGAGGGAATTTAAGGTCTTTTACACTCGGACCTCGTCGACATGCATT
AACGCTGAAGCGCTGGGGGCAAATTGTGTTACACTCGGACCTCGTCGACATGCATT
CCGTTAAGCTCACGCATGAGTTCACGGGTTACACTCGGACCTCGTCGACATGCATT
TTTGTTCTGGACCACGTCTGCCGAAAGCTTACACTCGGACCTCGTCGACATGCATT
GTCTTCATAGAACGAACAACGCACTACAAGACTTACACTCGGACCTCGTCGACATGCATT
AAATCTGAAGGAGTAGCATCCTTGATTTCACCTTACACTCGGACCTCGTCGACATGCATT
AGTTCTTTTCTTGTGCAGGGACATAAGTCACATTACACTCGGACCTCGTCGACATGCATT
TAGCACGTGGAACCCAATAGGCACACTTTTACACTCGGACCTCGTCGACATGCATT
GCATTTTGGGGTAAGTAACCACAAGTAGTGGCTTACACTCGGACCTCGTCGACATGCATT
TGAGTTGGTAGTCCCAAAATCTTTGAGGCTACTTACACTCGGACCTCGTCGACATGCATT
GACATTTGTGCGAACAGTATCTACACAAACTCTTACACTCGGACCTCGTCGACATGCATT
ATTGTTTGATGAAGCCAGCATCTGCAAGTGTTTACACTCGGACCTCGTCGACATGCATT
AACGTTCCGTGTACCAAGCAATTTCATGCTTTACACTCGGACCTCGTCGACATGCATT

AGTTGTTATCGACATAGCGAGTGTATGCCCTTACACTCGGACCTCGTCGACATGCATT
GGTCCAACCAGAAGTGATTGTACCCGCTTTACACTCGGACCTCGTCGACATGCATT
TAGGCGTGACAAGTTTCATTATGATCTTGCAGTTACACTCGGACCTCGTCGACATGCATT
GACTGTAAACTACATTCTTGGTGAAATGCAGCTTACACTCGGACCTCGTCGACATGCATT
TGTTCACGGCAGCAGTATACACCCCTTTACACTCGGACCTCGTCGACATGCATT
GCTTGTAAGGTTGCCCTGTTGTCCAGCTTACACTCGGACCTCGTCGACATGCATT
ATTTCCTTTGCACAGGTGACAATTTGTCCACTTACACTCGGACCTCGTCGACATGCATT
GTTTTCAAGCCAGATTCATTATGGTATTCGGCTTACACTCGGACCTCGTCGACATGCATT
CCCTTTGAGTGCGTGACAAATGTTTCACCTTTACACTCGGACCTCGTCGACATGCATT
TCGATGTACTGAATGGGTGATTTAGAACCAGCTTACACTCGGACCTCGTCGACATGCATT
GGAGAGTGCTAGTTGCCATCTCTTTTTGAGGTTACACTCGGACCTCGTCGACATGCATT
TGGGTATGTCACACTCATATGAGTTGTTGACATTACACTCGGACCTCGTCGACATGCATT
TATTGTTATAGCGGCCTTCTGTAAAACACGCTTACACTCGGACCTCGTCGACATGCATT
AAACAGTGCCAAAGATGTTAGTTAGCCACTGCTTACACTCGGACCTCGTCGACATGCATT
CAGCATCAGTAGTGTCAGCAATGTCTCTGTTACACTCGGACCTCGTCGACATGCATT
CAAAGGCAATAGTGCGACCACCCTTACGATTACACTCGGACCTCGTCGACATGCATT
CTTGGTCATAGACACTGGTAGAATTTCTGTGGTTACACTCGGACCTCGTCGACATGCATT
AGTCAACTGAACAACACCACCTGTAATGTAGTTACACTCGGACCTCGTCGACATGCATT
CTGATAACTAGCGCATATACCTGCACCTTACACTCGGACCTCGTCGACATGCATT
ATGTCAAGAATCTCAAGTGTCTGTGGATCACGTTACACTCGGACCTCGTCGACATGCATT
AAAGGCACATTTGGTTGCATTCATTTGGTGACTTACACTCGGACCTCGTCGACATGCATT
CCAATGTACACATAGTGCTTAGCACGTAATCTTACACTCGGACCTCGTCGACATGCATT
CATCCACGCACAATTCAATTAAAGGTGCTGATTACACTCGGACCTCGTCGACATGCATT
ACAGCAACCTGGTTAGAAGTATTTGTTCCTGGTTACACTCGGACCTCGTCGACATGCATT
GTGACAAGCTACAACACGTTGTATGTTTGCGATTACACTCGGACCTCGTCGACATGCATT
AAATTGTGAGTAACAACCAGTGGTGTGTACCCTTACACTCGGACCTCGTCGACATGCATT
TTGTAGCTTGTTTACCACACGTACAAGGTATCTTACACTCGGACCTCGTCGACATGCATT
ACAACAGCAAGTTGCAAACAAAGTGAACACCCTTACACTCGGACCTCGTCGACATGCATT
TACTATGGCAATCAAGCCAGCTATAAAACCTTACACTCGGACCTCGTCGACATGCATT
GAAGTGAATAGGACACGGGTCATCAACTACATTTACACTCGGACCTCGTCGACATGCATT
AGTTCCATCTCTAATTGAGGTTGAACCTCAACTTACACTCGGACCTCGTCGACATGCATT
GGTGCTGACATCATAACAAAAGGTGACTCCTTACACTCGGACCTCGTCGACATGCATT
TATCTAGTAATAGATTACCAGAAGCAGCGTGCTTACACTCGGACCTCGTCGACATGCATT
TAGTTTCTGGATTGAATGACCACATGGAACGCTTACACTCGGACCTCGTCGACATGCATT
ACGTGCCCGCCGAGGAGAATTAGTCTGTTACACTCGGACCTCGTCGACATGCATT
GTTCTTTTCACTGCACTTTGGAAAGTAACACCTTACACTCGGACCTCGTCGACATGCATT
TCCCATTTTTCAGTATAACCACCAATCTGGTTACACTCGGACCTCGTCGACATGCATT
AGTAGGTATAACCACAGCAGTTAAAACACCCTTTACACTCGGACCTCGTCGACATGCATT
TATGCAAAGTATGCCTACTTTTGCTCTGGTTTACACTCGGACCTCGTCGACATGCATT
GTTAACATTTGGGCCGACAACATGAAGACAGTTTACACTCGGACCTCGTCGACATGCATT
CCAGCAATACGAAGATGTCCACGAAGGATCATTACACTCGGACCTCGTCGACATGCATT
AGTAAGCAACTGAATTTTCTGCACCAAGTGACTTACACTCGGACCTCGTCGACATGCATT
ACTGTAATAGTTGTGTCCGTACCAGCTGCTTACACTCGGACCTCGTCGACATGCATT
TATCACCAACATCAGACACTAATGCCTGATTACACTCGGACCTCGTCGACATGCATT
AGTGTAGGCAATGATGGATTGACTAGCTACATTACACTCGGACCTCGTCGACATGCATT
GTCATGTTCAGAAATAGGACTTGTTGTGCCATTTACACTCGGACCTCGTCGACATGCATT

ATGTGATGTTGATATGACATGGTCGTAACAGCTTACACTCGGACCTCGTCGACATGCATT
GTTGAGTTTGAAGGCATCTATGCTATTCTTGGTTACACTCGGACCTCGTCGACATGCATT
CACATGTCTTGGACAGTAAACTACGTCATCAATTACACTCGGACCTCGTCGACATGCATT
GTCCTCTGACTTCAGTACATCAAACGAATTTGTTACACTCGGACCTCGTCGACATGCATT
TGAACAACATCACCCACTATATATGGAGCATTACACTCGGACCTCGTCGACATGCATT
CTTATTTAAGGCTCCTGCAACACCTCCTTACACTCGGACCTCGTCGACATGCATT
CACAGGCGAACTCATTTACTTCTGTACCGATTACACTCGGACCTCGTCGACATGCATT
AACGGTCAAAGAGTTTTAACCTCTCTTCCGTGTTACACTCGGACCTCGTCGACATGCATT
GTGTTGAACCTTTCTACAAGCCGCATTAATCTTACACTCGGACCTCGTCGACATGCATT
ACCAAGGTCACGGGGTGTCATGTTTTCAACTTACACTCGGACCTCGTCGACATGCATT
CGCAGGCAAGATTATCCATTCCCTGCTTACACTCGGACCTCGTCGACATGCATT
ATTTGACAGCAGAATTGGCCCTTAAAGCTGTTTACACTCGGACCTCGTCGACATGCATT
TCCAGTTGGTAATTCCATATGGTGCATGTAACTTACACTCGGACCTCGTCGACATGCATT
GGCAGTACAGACAACACGATGCACCACTTACACTCGGACCTCGTCGACATGCATT
CCCACTTTAAGTGGTCCATTAGTAGCTATGTATTACACTCGGACCTCGTCGACATGCATT
ACAAAGCACTCGTGGACAGCTAGACACCTTACACTCGGACCTCGTCGACATGCATT
AACGTGTGTCAGGGCGTAAACTTTCATAAGCATTACACTCGGACCTCGTCGACATGCATT
AACTGTTATTGCCTGACCAGTACCAGTGTTTACACTCGGACCTCGTCGACATGCATT
GTTGTACCACAAGTTACTTGTACCATACAACCTTACACTCGGACCTCGTCGACATGCATT
GCTTTAGGGTTACCAATGTCGTGAAGAACTGGTTACACTCGGACCTCGTCGACATGCATT
ACACTCAAGAACGTCTTTCTGTATGGTAGGATTTACACTCGGACCTCGTCGACATGCATT
GAAGCCAATCCATGCACGTACATGTCTTATTACACTCGGACCTCGTCGACATGCATT
AGGAATGAACCCTTAATAGTGAAATTGGGCCTTTACACTCGGACCTCGTCGACATGCATT
TGTCATTAGTATAACTACCACCACGCTGGTTACACTCGGACCTCGTCGACATGCATT
AAGTCACCATTAGTTGTGCGTAATATCGTGCCTTACACTCGGACCTCGTCGACATGCATT
AAGATGGATGGAACCATTCTTCACTGTAACACTTACACTCGGACCTCGTCGACATGCATT
TAAAGCTCACAAGTAGCGAGTGTTATCAGTGCTTACACTCGGACCTCGTCGACATGCATT
ACCTCCAAGAGCTAGCTTTTGTTGTATAAACCTTACACTCGGACCTCGTCGACATGCATT
CCTCTACAGTGTAACCATTTAAACCCTGACTTACACTCGGACCTCGTCGACATGCATT

2. Synthesis:

tert-Butyl ((_S_)-6-azido-1-(((2_S_,3_R_)-1,3-dihydroxy-(_E_)-octadec-4-ene-2-yl)amino)-1-oxohexane-2-yl)carbamate 1

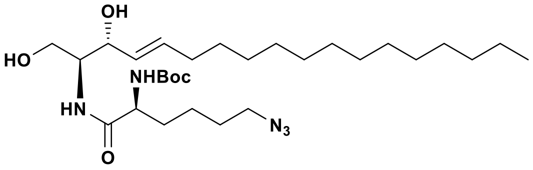

1

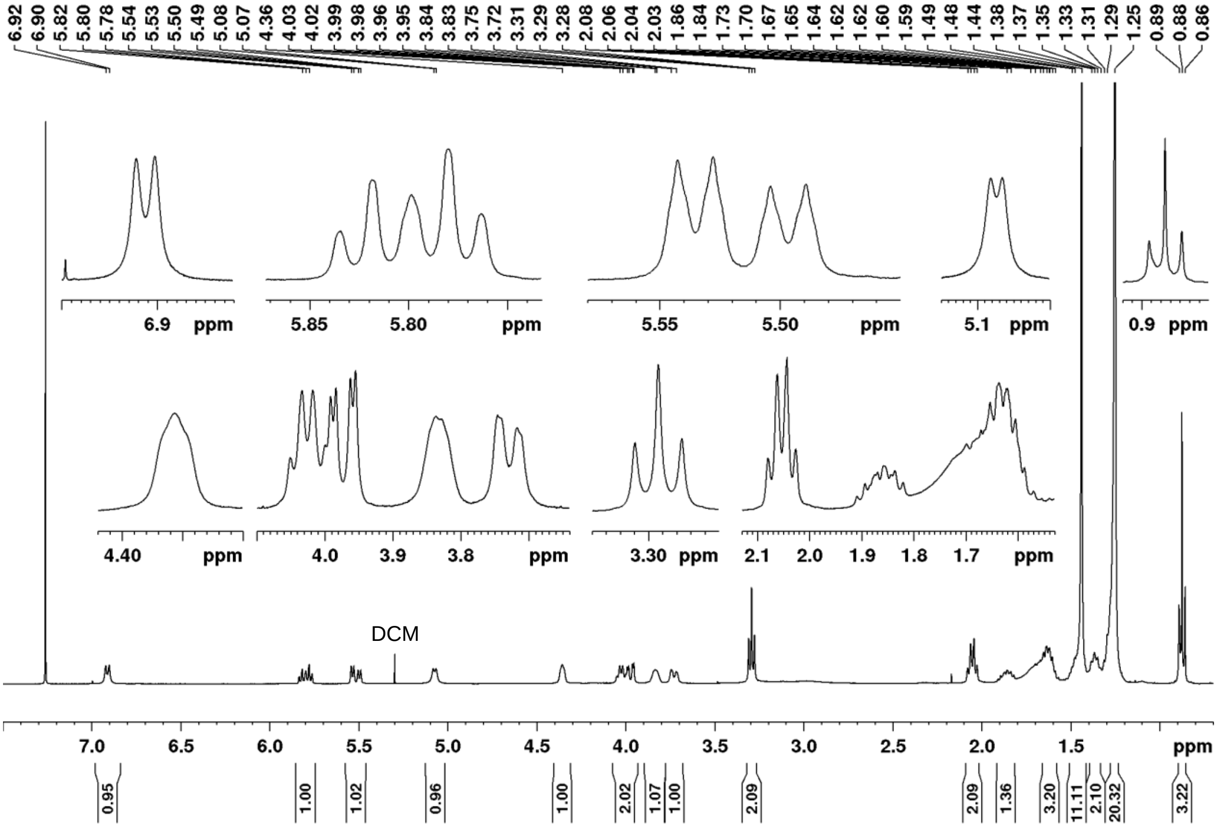

Figure S1: ^{1}H NMR spectrum of **1** (400 MHz, $CDCl_3$).

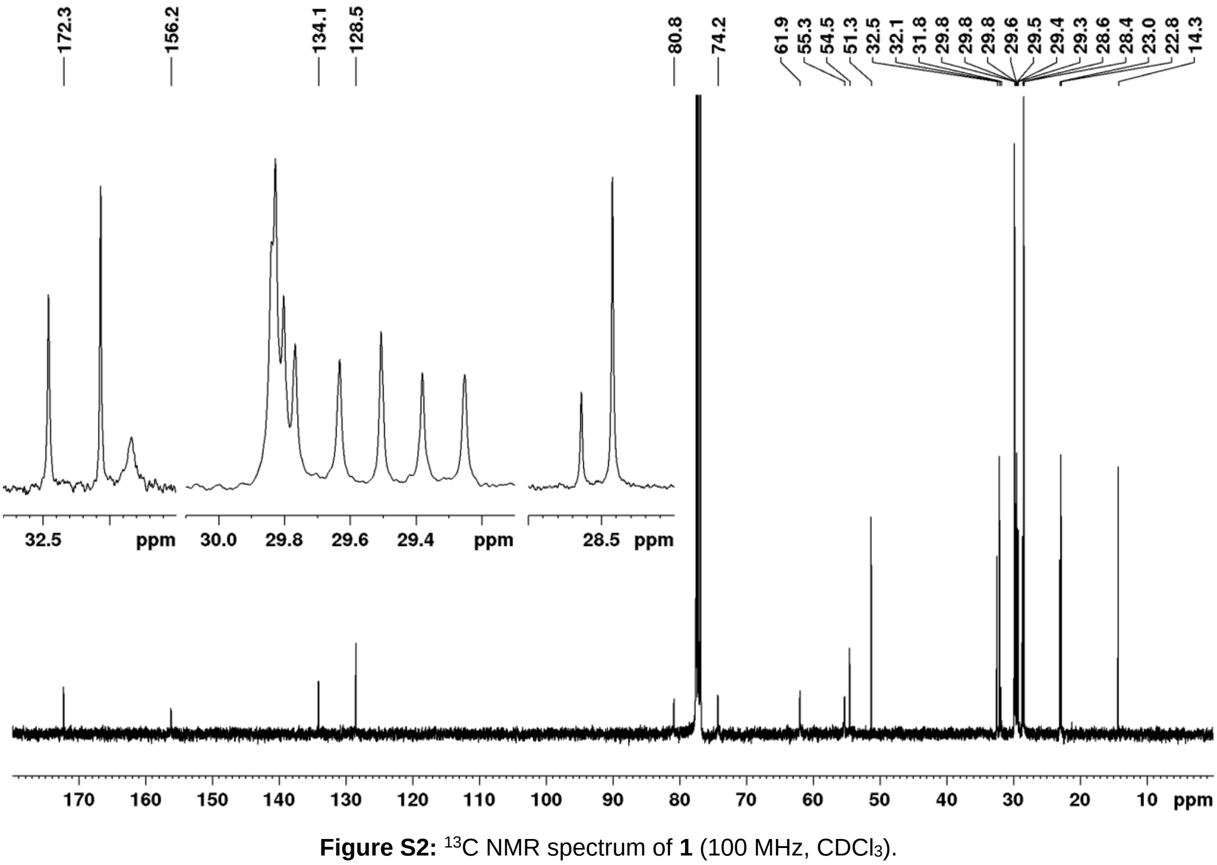

Figure S2: ^{13}C NMR spectrum of **1** (100 MHz, $CDCl_3$).

(*S*)-2-Amino-6-azido-*N*-((2*S*,3*R*)-1,3-dihydroxy-(*E*)-octadec-4-ene-2-yl)hexanamide 2

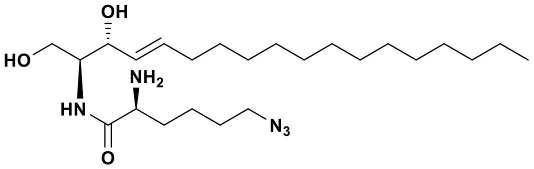

2

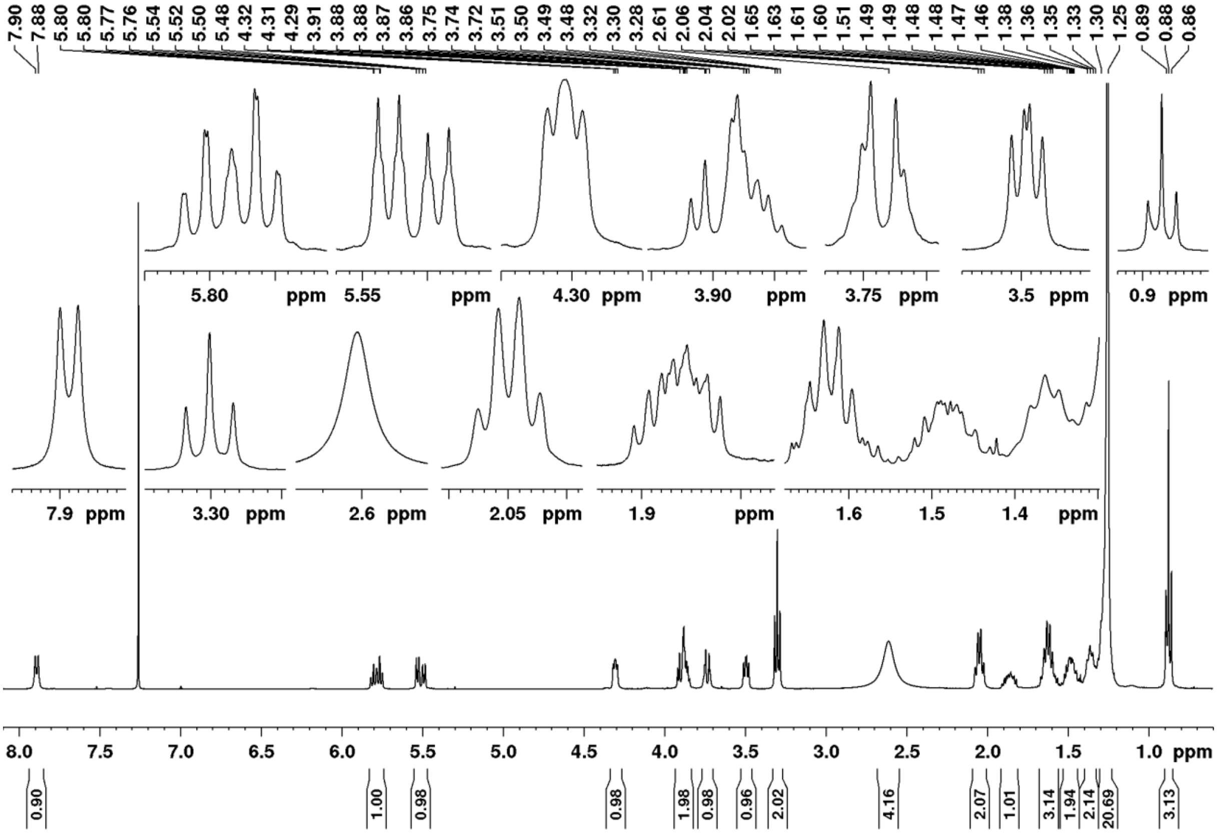

Figure S3: ^{1}H NMR spectrum of **2** (400 MHz, $CDCl_3$).

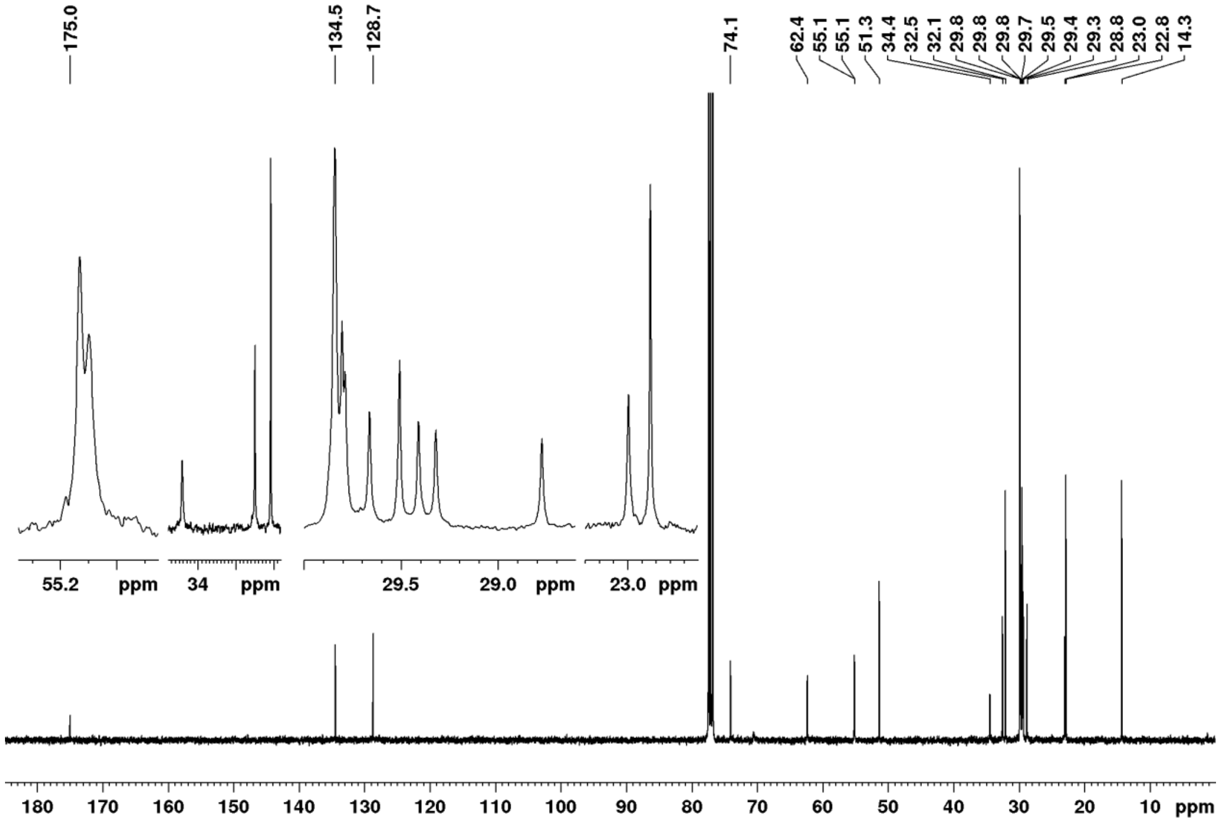

Figure S4: ^{13}C NMR spectrum of **2** (100 MHz, $CDCl_3$).
